# Diversified Application of Barcoded PLATO (PLATO-BC) Platform for Identification of Protein Interactions

**DOI:** 10.1016/j.gpb.2018.12.010

**Published:** 2019-09-05

**Authors:** Weili Kong, Tsuyoshi Hayashi, Guillaume Fiches, Qikai Xu, Mamie Z. Li, Jianwen Que, Shuai Liu, Wei Zhang, Jun Qi, Netty Santoso, Stephen J. Elledge, Jian Zhu

**Affiliations:** 1 Department of Microbiology and Immunology, Department of Biochemistry and Biophysics, University of Rochester Medical Center, Rochester, NY 14642, USA; 2 Division of Genetics, Brigham and Women's Hospital, Howard Hughes Medical Institute, Department of Genetics, Harvard Medical School, Boston, MA 02115, USA; 3 Department of Medicine, Columbia University Medical Center, New York, NY 10032, USA; 4 Department of Chemistry, College of Science and Mathematics, University of Massachusetts Boston, Boston, MA 02125, USA; 5 Dana-Farber Cancer Institute, Harvard Medical School, Boston, MA 02115, USA; 6 Department of Pathology, Ohio State University Wexner Medical Center, Columbus, OH 43210, USA

**Keywords:** Barcoded PLATO, Protein interaction, Ubiquitin-binding protein, Bromodomain inhibitor JQ1, Zika virus

## Abstract

Proteins usually associate with other molecules physically to execute their functions. Identifying these interactions is important for the functional analysis of proteins. Previously, we reported the parallel analysis of translated ORFs (PLATO) to couple ribosome display of full-length ORFs with affinity enrichment of mRNA/protein/ribosome complexes for the “bait” molecules, followed by the deep sequencing analysis of mRNA. However, the sample processing, from extraction of precipitated mRNA to generation of DNA libraries, includes numerous steps, which is tedious and may cause the loss of materials. **Barcoded PLATO** (PLATO-BC), an improved platform was further developed to test its application for **protein interaction** discovery. In this report, we tested the antisera-antigen interaction using serum samples from patients with inclusion body myositis (IBM). Tripartite motif containing 21 (TRIM21) was identified as a potentially new IBM autoantigen. We also expanded the application of PLATO-BC to identify protein interactions for JQ1, single ubiquitin peptide, and NS5 protein of **Zika virus**. From PLATO-BC analyses, we identified new protein interactions for these “bait” molecules. We demonstrate that Ewing sarcoma breakpoint region 1 (EWSR1) binds to JQ1 and their interactions may interrupt the EWSR1 association with acetylated histone H4. RIO kinase 3 (RIOK3), a newly identified **ubiquitin-binding protein**, is preferentially associated with K63-ubiquitin chain. We also find that Zika NS5 protein interacts with two previously unreported host proteins, par-3 family cell polarity regulator (PARD3) and chromosome 19 open reading frame 53 (C19orf53), whose attenuated expression benefits the replication of Zika virus. These results further demonstrate that PLATO-BC is capable of identifying novel protein interactions for various types of “bait” molecules.

## Introduction

Proteins are the workhorses that control many biological processes. They recognize or are recognized by other molecules through physical interactions to achieve proper biological functions. Characterization of protein-binding events remains an important matter to understand protein functions. A variety of proteomic tools have been developed to allow the massive identification of protein interactions for a “bait” molecule [1], [2]. Identity of protein/peptide can be directly analyzed by mass spectrometers or by pre-arrayed protein microarrays. However, such direct detection of protein/peptide signal usually requires enormous efforts. Instead, protein/peptide-corresponding nucleotide signal is relatively easy to identify, due to the recent advances of high-throughput sequencing technologies. To “pair” protein/peptide with its coding sequence, a vector is usually needed to express and display the protein/peptide. The commonly used vectors include phages, yeasts, and ribosomes [3].

We recently developed the parallel analysis of translated ORFs (PLATO), which is a method to display full-length proteins on ribosomes *in vitro* and analyze the enriched mRNA species through the high-throughput DNA sequencing [4], [5]. PLATO has been demonstrated to perform protein interaction screens against the human ORFeome for diverse baits, including proteins, antibodies, and small-molecule compounds. For PLATO, the 3′ termini of affinity-enriched ORF mRNAs have to be recovered and further processed to DNA libraries for deep sequencing. This strategy would not only retain stoichiometric correlation between tag counts and transcript abundance, but also lessen the adverse impact of RNA degradation. However, it requires a laborious procedure including multiple steps: (i) chemical fragmentation of enriched mRNAs to generate the short species; (ii) reverse transcription of the mRNA fragments containing the 3′ end of ORFs using a primer recognizing the common region (from the vector) at the downstream of ORF mRNAs; (iii) polyadenylation of the cDNAs containing the 3′ end of ORFs; and (iv) addition of the sample barcodes and sequencing adaptors to the polyadenylated cDNA species by two-step PCR amplifications. To simplify the sample processing of PLATO, barcodes were added at the 3′ end of each ORF [6]. In this report, we expanded the diversified applications of barcoded PLATO (PLATO-BC) and further demonstrated that it is an improved method useful for versatile applications of protein interaction discovery.

## Materials and methods

### PLATO-BC platform

We used the PLATO-BC library as previously described with slight modifications [5], [6]. For PLATO assay, the human ORFeome v5.1 pRD-DEST plasmid DNA (Catalog No. OHS5177, Dharmacon, Lafayette, CO) was linearized with PI-SceI and then was *in vitro* transcribed using the T7 high yield kit (Catalog No. E2040S, New England Biolabs, Ipswich, MA). The RNA was purified using RNA cleanup kit (Catalog No. 74204, Qiagen, Germantown, MD), and 2.5 μg was used for a 100-μl *in vitro* translation reaction. A total of 12.5 μl of the *in vitro* translation reaction is diluted in 85.5 μl of selection buffer. The different bait molecules were immobilized using different reagents. (1) Immobilization of patient antibodies. ∼2.0 μg of immunoglobulin from each patient sample or healthy donor was incubated with Dynabeads protein A- and G-coated magnetic beads (Catalog No. 88802, Thermo Fisher Scientific, Waltham, MA) (a 1:1 mixture) at 4 °C, rotating end-over-end overnight. (2) Immobilization of biotinylated molecules. Biotinylated JQ1 (synthesized in house) or ubiquitin (Ub) (Catalog No. UB-570, BostonBiochem, Cambridge, MA) was immobilized on Dynabeads MyOne streptavidin T1 magnetic beads (Catalog No. 65601, Thermo Fisher Scientific) by incubation in 1× PBST at 4 °C overnight. Equal moles of free biotin were immobilized as well. Generally, we immobilized 20 μmol of biotinylated molecules per 1 ml of beads and used 25 μl of beads. (3) Immobilization of V5-tagged Zika virus (ZIKV)-NS5 protein. ZIKV-NS5 cDNA was cloned into the pcDNA-DEST40 vector (Catalog No. 12274015, Thermo Fisher Scientific). pcDNA-DEST40 vector containing ZIKV-NS5 or a short flag peptide (DYKDDDDK) was transfected into HEK293T cells. At 48 h post transfection, cells were harvested and lysed in 1× RIPA buffer [50 mM Tris-HCl (pH 7.4), 150 mM NaCl, 1% NP-40, 0.25% sodium deoxycholate, and 1 mM EDTA]. The lysate was centrifuged at 4000*g* for 10 min at 4 °C and the supernatant was aspirated. About 2 μg of an anti-V5 antibody (Catalog No. R960-25, Thermo Fisher Scientific) was incubated with the supernatant overnight at 4 °C. 30 μl of Dynabeads protein A- and G-coated magnetic beads was added for a further incubation at 4 °C for 2 h. Magnetic beads were collected on a magnetic stand (Invitrogen) for 30 s, and the supernatant was removed. Beads were re-suspended and rinsed with 1× washing buffer [50 mM Tris acetate, 150 mM NaCl, 50 mM magnesium acetate, 0.5% Tween 20 (pH 7.5), DEPC treated] for 5 times. Beads were subsequently blocked in 1× selection buffer [2.5 mg/ml heparin, 1% (w/v) BSA, and 100 μg/ml yeast tRNA in 50 mM Tris acetate and 150 mM NaCl (pH 7.5), DEPC treated] for 2 h at 4 °C and further subjected to the PLATO assay [5]. Barcodes were analyzed by single-end deep sequencing [6].

### Cell culture

The HFF-1 skin fibroblast cells (Catalog No. SCRC-1041, ATCC, Manassas, VA), MAGI cells (Catalog No. 1470, NIH AIDS reagent program, Germantown, MD) and HEK293T embryonic kidney cells (Catalog No. CRL-3216, ATCC), were cultured in Dulbecco’s modified Eagle’s medium (DMEM) supplemented with 10% fetal bovine serum (FBS), penicillin (100 U/ml), and streptomycin (100 μg/ml).

### Constructs

The NS5 ORF was amplified with PCR from the cDNA of the contemporary ZIKV PRVABC59 strain and then cloned into pDNOR221 vector (Catalog No. 12536017, Thermo Fisher Scientific) through gateway BP reaction. Bromodomain 1 (BD1) and bromodomain 2 (BD2) fragments were amplified with PCR from bromodomain-containing protein 4 (BRD4) plasmid and then cloned into pDNOR221 vector. *Tripartite motif containing 21* (*TRIM21*), *Ewing sarcoma breakpoint region 1* (*EWSR1*), *eyes absent homolog 3* (*EYA3*), *RNA binding motif protein 14* (*RMB14*), *coiled-coil domain containing 124* (*CCDC124*), *par-3 family cell polarity regulator* (*PARD3*), *RING1 and YY1 binding protein* (*RYBP*), *chromosome 19 open reading frame 53* (*C19orf53*), *inhibitor growth of protein 2* (*ING2*) and *RIO kinase 3* (*RIOK3*) were picked up from MISSION TRC3 human LentiORF library (Sigma, St. Louis, MO) and then cloned into destination vector including pcDNA-DEST40, pET-DEST42 (Catalog No. 12276010, Thermo Fisher Scientific), pEZY-Myc (Catalog No. 18701, Addgene, Watertown, MA), and pEZY-FLAG (Catalog No. 18700, Addgene). HA-Ub-wt (Catalog No.17608), HA-Ub-K48 (Catalog No.17605), and HA-Ub-K63 (Catalog No.17606) were purchased from Addgene. The clones were confirmed by Sanger sequencing.

### Antibodies

Mouse anti-Myc (Catalog No. sc40), anti-TRIM21 (Catalog No. sc25351) and IgG (Catalog No. sc2025) were obtained from Santa Cruz Biotechnology, Dallas, TX. Anti-Flag (Catalog No. 2368) was purchased from Cell Signaling Technology, Danvers, MA. Anti-V5 (Catalog No. R960-25) and anti-histone H4ac (pan-acetyl) (Catalog No. 39926) were purchased from Thermo Fisher Scientific and Active Motif, Carlsbad, CA, respectively.

### Proteins and peptides

ORF sequences for EWSR1, EYA3, RMB14, BD1, and BD2 were cloned into pET-DEST42 vector, which was transformed into Rosetta™ (DE3) *E. coli* strain for protein expression and purification. Plasmid-transformed Rosetta™ (DE3) bacteria cells were incubated in LB media at 37 °C until the optical density (OD) at 600 nm reached ∼0.6. Protein expression was induced by treating cells with 0.1 mM IPTG for 16 h at 16 °C. Cells were harvested and re-suspended in 1× lysis buffer [50 mM NaH_2_PO_4_ (pH 8.0), 500 mM NaCl] on ice. Eight mg lysozyme was added and incubated on ice for 30 min and then cells were lysed by brief sonication. Cell debris were removed by centrifugation at 12,000*g* for 10 min at 4 °C. The supernatant was loaded onto 1 ml HisTrap column of His-tagged fusion protein purification system (Catalog No. 88225, Thermo Fisher Scientific) which was washed with the binding buffer. His-tagged proteins were eluted with the elution buffer. Protein concentration was measured by using the Bio-Rad protein assay kit. Biotinylated histone H4 peptides (Catalog No. 120029) and biotinylated acetylated histone H4 peptides (Catalog No. 120047) were purchased from EpiCypher, Durham, NC.

### Patient samples

IBM serum and healthy donor (HD) samples for this study were previously described [5], [7]. All patient samples were collected after informed written consent was obtained and under protocols approved by the Partners Human Research Committee Institutional Review Board overseeing Brigham and Women's Hospital human research activities.

### Protein pull-down assays

5 μg of His-tagged proteins (EWSR1, EYA3, RBM14, BD1, BD2, and RIOK3) was incubated with 1 μg of biotinylated molecules (histone H4, Ub, or JQ1) in the binding buffer [50 mM Tris (pH7.5), 150 mM NaCl, 0.1% NP-40] for 2 h at 4 °C. The mixture was further incubated with 30 μl of streptavidin-coated beads (Catalog No. 65601, Thermo Fisher Scientific) for 2 h with rotation at 4 °C. The beads were washed with binding buffer for four times.

For the protein co-immunoprecipitation, HEK293T cells were seeded in 10-cm dishes 24 h prior to the transfection. NS5-V5-expressing vector and hit genes expressed in pEZY vector were transfected into HEK293T cells using TurboFect reagents (Catalog No. R0531, Thermo Fisher Scientific). At 48 h post transfection, cells were washed with ice-cold 1× PBS and lysed in 1× RIPA buffer containing 2 mM PMSF and protease inhibitors. Cell lysate was centrifuged at 13,000*g* for 10 min at 4 °C. The aspirated supernatant was divided into halves, and incubated with anti-V5 or anti-Myc antibodies or mouse IgG. At 16 h post incubation at 4 °C, 30 μl of a 50% slurry of protein A- and G-sepharose were added for incubation for another 2 h at 4 °C. Beads were collected by centrifugation and washed with 1× RIPA buffer for three times. Protein samples were eluted from beads and boiled in the 2× SDS loading buffer, and analyzed by immunoblotting. Protein samples were separated by SDS-PAGE, and transferred to PVDF. Blots were blocked with 5% skimmed milk in PBS and probed with anti-V5, anti-Flag or anti-Myc primary antibodies followed by anti-mouse HRP-conjugated secondary antibodies (Catalog No. 6265620, Thermo Fisher Scientific). Protein bands were visualized with ECL Plus chemiluminescence reagent (Catalog No. 32132, Thermo Fisher Scientific).

### Preparation of ZIKV

The PRVABC59 ZIKV strain was obtained from Dr. Stephen Dewhurst’s lab at University of Rochester medical center. The virus was amplified in Vero cells according to previously published protocol [8]. The titer of ZIKV was measured by median tissue culture infectious dose (TCID_50_) in Vero cells.

### siRNA knockdown and ZIKV infection

All siRNAs were purchased from Ambion, Austin, TX (Table S1). The control siRNA is a pool of 4 Silencer Negative Control siRNAs (Catalog Nos. AM4611, AM4613, AM4615, and AM4641). Gene-specific siRNAs were as follows: *AXL receptor tyrosine kinas* (*AXL*) siRNAs (Catalog Nos. s1846 and s1847); *CCDC124* siRNAs (Catalog Nos. s41756 and s225505); *C11orf46* siRNAs (Catalog Nos. s42387 and s42388); *C19orf53* siRNAs (Catalog Nos. s26334 and s226241); *interferon-induced transmembrane protein 3* (*IFITM3*) siRNAs (Catalog Nos. s195034 and s195035); *ING2* siRNAs (Catalog Nos. s7431 and s7433); *PARD3* siRNAs (Catalog No. s32126 and s32128); *RYBP* siRNAs (Catalog Nos. s23812 and s23813); *ZC3H15* siRNAs (Catalog Nos. s31666 and s31667). siRNAs targeting several genes (*CCDC124*, *C19orf53*, *PARD3*, *RYBP*, *C11orf46*, *ZC3H15*, *ING2*, *IFITM3*, and *AXL*) were transfected into the HFF-1 cells in triplicate at 20 nM by using 0.32% Oligofectamine (Catalog No. 12252011, Thermo Fisher Scientific) in 384-well plates. At 72 h post transfection, the medium was removed and the cells were infected with ZIKV at ∼ 0.2 to 0.3 multiplicity of infection (MOI) in 40 μl complete media. At 48 h post infection, the media were removed and cells were fixed with 4% formalin. For cell staining, an anti-*Flavivirus* group antigen primary antibody (Catalog No. MAB10216, Millipore Sigma, Burlington, MA) and an Alexa Fluor 488 goat anti-mouse secondary antibody (Catalog No. A11001, Invitrogen) were used. The nucleus was stained with DAPI. Cells were imaged on Cytation 5 cell imaging multi-mode reader (Catalog No. Cytation 5, BioTek, Winooski, VT) and analyzed using Gen5 software. These experiments were performed in the BSL-2+ biological safety cabinets.

### Real-time qPCR

Total RNA was extracted from eluted mRNA samples or the siRNA-transfected HFF-1or MAGI cells by using the RNeasy kit (Qiagen), and 1 µg of RNA was reversely transcribed by using the iScript™ cDNA Synthesis Kit (Catalog No. 1708890, Bio-Rad, Hercules, CA). Real-time PCR assay was performed by using the SYBR Premix (Catalog No. 4106212, Bio-Rad). The primers of different genes for qPCR are listed in Table S1. The PCR reactions were run on an Bio-Rad CFX connect qPCR machine under the following conditions: 95 °C for 10 min, followed by 40 cycles of 95 °C for 15 s and 60 °C for 1 min. Relative fold of gene expression was normalized to the *GAPDH* or *BC-*Input control. Fold change of gene expression was calculated using the formula: 2^(ΔCT of gene−ΔCT of GAPDH)^ or 2^(ΔCT of gene−ΔCT of BC-Input)^.

### Statistical analysis

Statistical analysis and graphical presentation was performed using GraphPad Prism 6 software. *P* values between the groups was compared using one-way analysis of variance (ANOVA)*. P* < 0.05 is considered statically significantly. *P < 0.05; **P < 0.01; ***P < 0.001.

## Results

### PLATO-BC for identifying antibody-protein interaction

**Figure 1:**
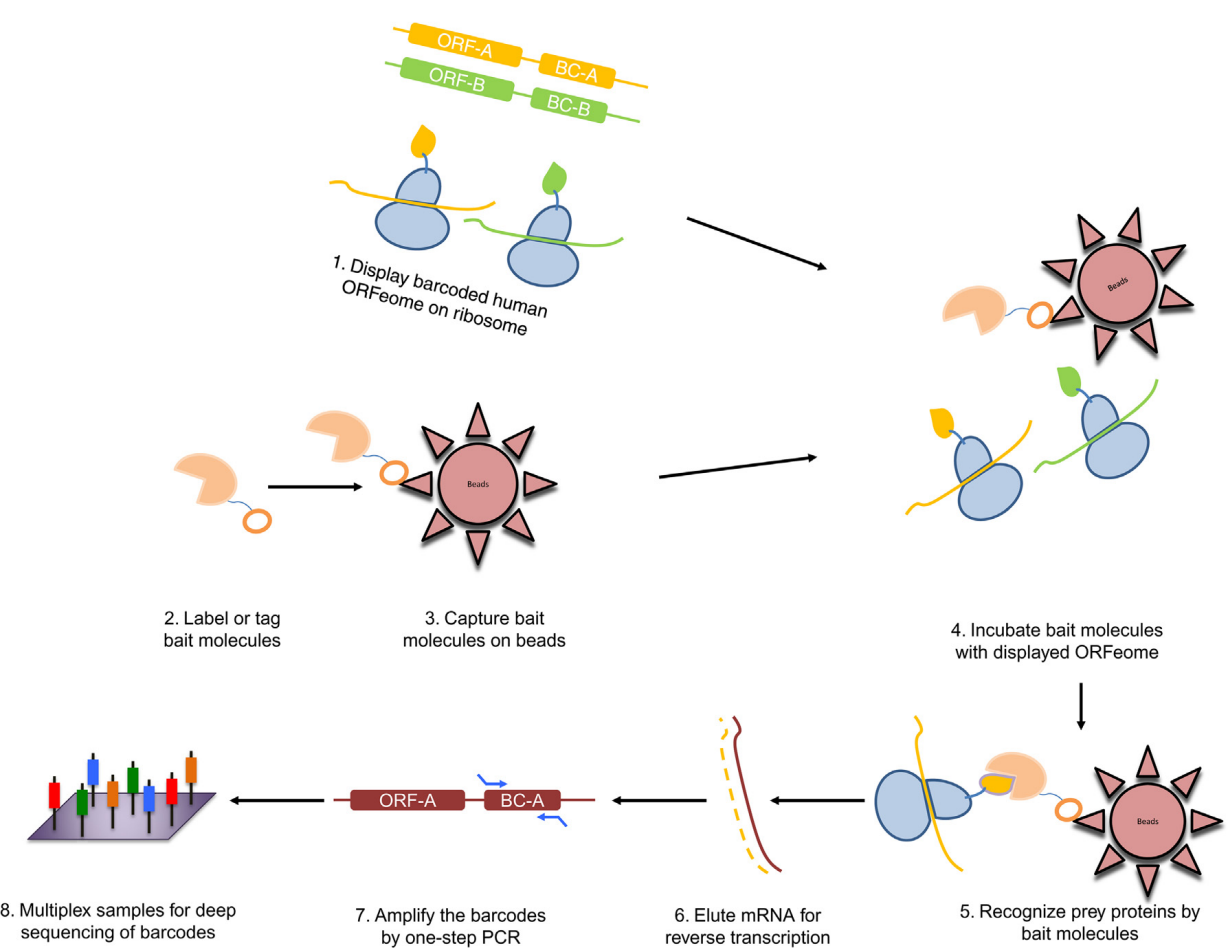
**Scheme of PLATO-BC assay to identify protein binders for “bait” molecules** Briefly, labeled or tagged “bait” molecules, including antisera, drugs, peptides, or proteins of the interest, are immobilized on magnetic beads. Barcoded ORFeome library is *in vitro* transcribed and translated to create the mRNA-ribosome-polypeptide ternary complexes, which are further incubated with immobilized “bait” molecules. Magnetic beads are precipitated and washed. The bound ribosome complexes are interrupted, and the associated mRNAs are eluted and reversely transcribed to produce cDNA templates. The barcode region is amplified by PCR to generate the DNA library that is analyzed by deep sequencing. PLATO, parallel analysis of translated ORFs; PLATO-BC, barcoded PLATO.

**Figure 2:**
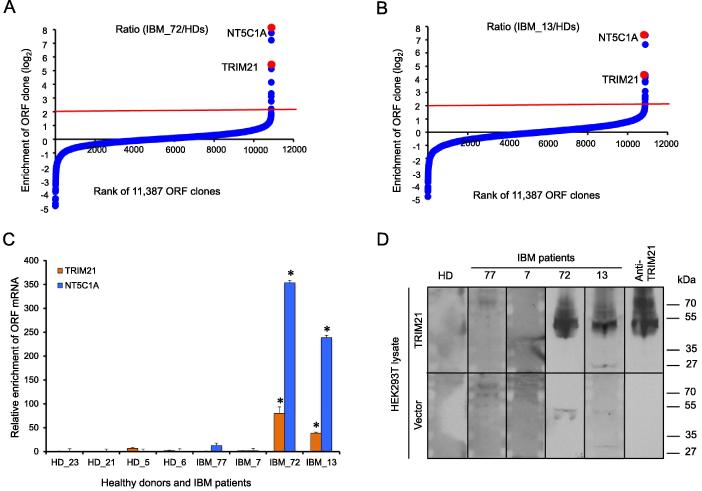
**Identification of protein binders for IBM antigen discovery by using PLATO-BC assay** Rank of ORF enrichment from the PLATO-BC assays of antisera samples from IBM patient 72 (**A**) and patient 13 (**B**) relative to HDs, respectively. ORFs were ranked according to the ratio of IBM/HDs (from the smallest to the largest). The red line shows the cut-off value. A known IBM autoantigen, NTSC1A, and a new one, TRIM21, were ranked as top hits (red dots) for both IBM patients (IBM_72 and IBM_13). **C.** mRNA enrichment of known (NT5C1A) and new (TRIM21) autoantigens from the PLATO-BC assays of antisera samples from IBM patients relative to HDs. mRNA level was quantified by RT-qPCR. Relative enrichment of NT5C1A and TRIM21 was normalized to HDs. Data are presented as mean ± SD (*n* = 3). *, *P <* 0.05, ANOVA. **D.** Validation of the new IBM autoantigen TRIM21 by immunoblotting. pcDNA-DEST40-TRIM21 was transiently expressed in HEK293T cells. Cell lysate was prepared and separated by SDS-PAGE. Either a commercial anti-TRIM21 antibody, or the antisera from IBM patients or HDs, was used for immunoblotting of exogenously expressed TRIM21. IBM, inclusion body myositis; HD, healthy donor; TRIM21, Tripartite motif-containing protein 21; NT5C1A, 5′-nucleotidase, cytosolic 1A.

The procedure of PLATO-BC to identify protein interaction is quite similar as previously described, except that the 30-mer barcodes, instead of 3′ ends of each ORF clone, are amplified by PCR and sequenced to recognize the ORF identity (Figure 1). We initially used the antisera samples with known antigens to test the PLATO-BC method. Serum antibodies from patients with IBM or HD were immobilized on protein A/G magnetic beads, and analyzed by PLATO-BC method for identification of IBM autoantigens (Figure 2A and B). We were able to identify a known one, the cytosolic 5′-nucleotidase 1A protein (NT5C1A), and also a new one, the tripartite motif containing protein TRIM21 (Figure 2C). We then exogenously expressed a V5-tagged TRIM21 protein in HEK293T cells, which was recognized by the IBM antisera samples in an immunoblotting assay (Figure 2D). This result validates that PLATO-BC is able to identify antigens that bind to the antisera samples.

### PLATO-BC for identifying compound-protein interaction

Small-molecule compounds usually recognize multiple protein targets, due to their binding flexibility. Identification of compound-protein interactions is critical for current drug development, especially for understanding of drug side effects and also for drug repurposing [9]. We test whether PLATO-BC is capable of identifying protein targets for small-molecule compounds. JQ1 is a methyl-triazolo bromodomain and extraterminal domain inhibitor (BETi). It binds to a set of bromodomain-containing proteins (BRDs), including BRD2, by binding competitively to bromodomains at the acetyl-lysine recognition motifs.

**Figure 3:**
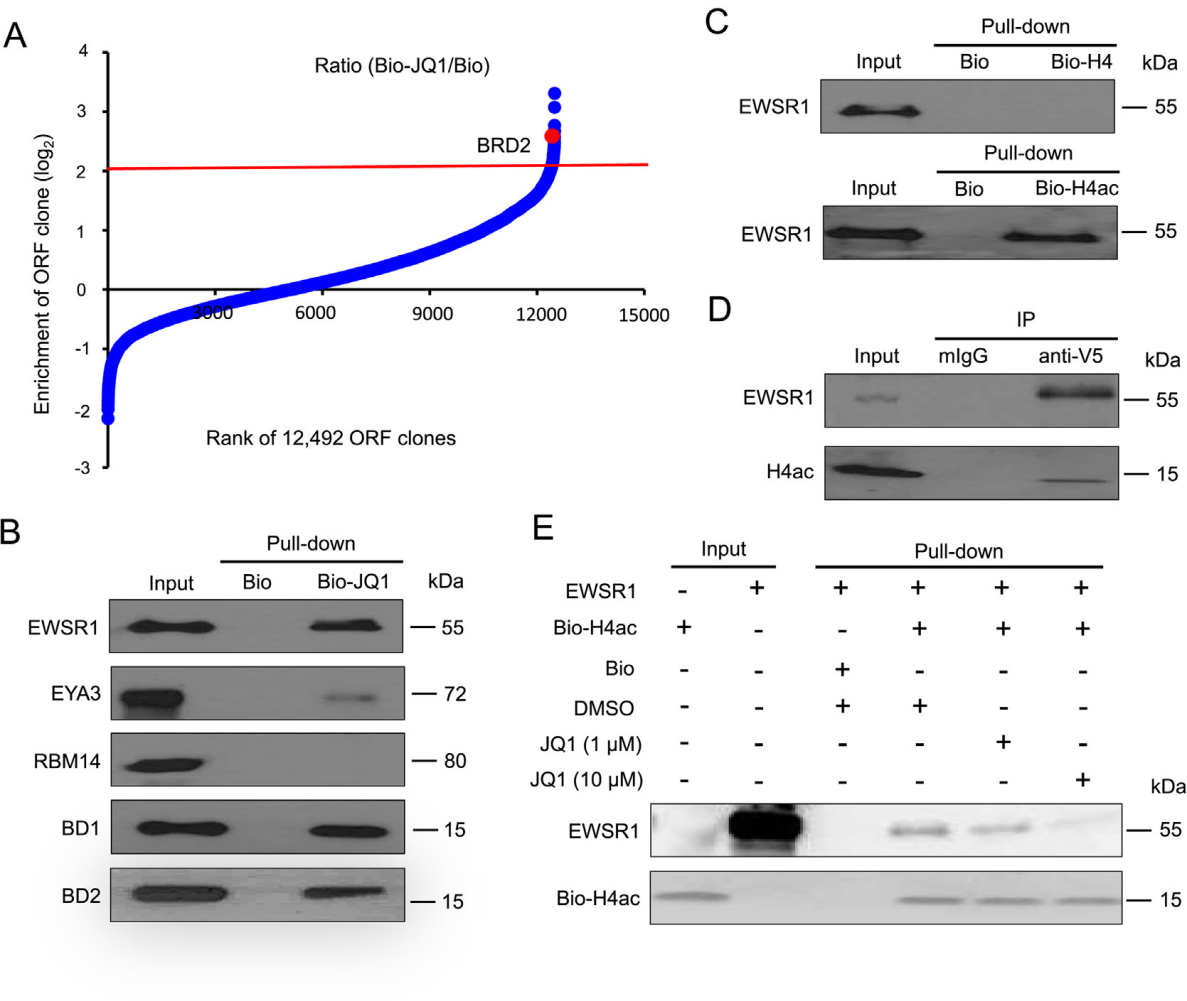
**Identification of protein binders for BETi JQ1 by using PLATO-BC assay** **A.** Rank of ORF enrichment from the PLATO-BC assays for biotinylated JQ1 (Bio-JQ1) relative to free biotin (Bio). 12,492 ORFs were ranked according to the ratio of Bio-JQ1/Bio (from the smallest to the largest). The red line shows the cut-off value. A known protein target of JQ1, BRD2, was ranked as a top hit (red dot). **B.** Hit validation of JQ1 protein targets identified from the PLATO-BC assay. pET-DEST42-EWSR1/EYA3/RBM14/BD1/BD2 was transformed into the *E. coli* DE3 strain. V5-His6-tagged EWSR1/EYA3/RBM14/BD1/BD2 proteins were purified and subjected to protein pull-down assays for Bio-JQ1 or Bio. Protein samples were separated by SDS-PAGE and analyzed by immunoblotting using an anti-V5 antibody. **C.** Interaction of EWSR1 with H4ac peptide *in vitro*. V5-His6-tagged EWSR1 protein was purified and subjected to protein pull-down assays for biotinylated, acetylated or non-acetylated H4 (Bio-H4ac or Bio-H4) peptide. Protein samples were separated by SDS-PAGE and analyzed by immunoblotting using an anti-V5 antibody. **D.** Interaction of EWSR1 with H4ac in cells. pcDNA-DEST40-EWSR1 was transiently transfected in HEK293T cells. Cell lysate was prepared and subjected to the protein immunoprecipitation assays for V5-His6-tagged EWSR1 using an anti-V5 mouse antibody or a mouse IgG (mIgG) control. Protein samples were separated by SDS-PAGE and analyzed by immunoblotting using an anti-H4ac antibody. **E.** JQ1 interrupts the interaction of EWSR1 with H4ac peptide *in vitro*. V5-His6-tagged EWSR1 protein was purified and incubated with JQ1 (at 1 μM or 10 μM) or DMSO, prior to the protein pull-down assays for Bio-H4ac or free biotin (Bio). Protein samples were separated by SDS-PAGE and analyzed by immunoblotting using an anti-V5 antibody or HRP-conjugated streptavidin to detect EWSR1 or H4ac respectively. BETi, bromodomain and extraterminal domain inhibitor; BRD2, bromodomain containing 2; H4ac, acetylated histone H4; EWSR1, Ewing sarcoma breakpoint region 1; EYA3, EYA transcriptional coactivator and phosphatase 3; RBM14, RNA-binding protein 14; BD1, bromodomain 1; BD2, bromodomain 2.

We used PLATO-BC to identify previously undiscovered protein binding partners for JQ1. We immobilized the biotinylated derivative of JQ1 (Bio-JQ1) or just biotin (Bio) on streptavidin-coated magnetic beads, which was subjected to PLATO-BC analysis. The ORF clones were ranked according to the ratio of enrichment (Bio-JQ1/Bio) (Figure 3A). We identified 122 top-ranked protein candidates (cutoff, log_2_ = 2), and BRD2 was one of the top hits with 6-fold enrichment (Figure 3A, Table S1). Surprisingly, the top two hits, EWSR1 and EYA3 both play a role in the development of Ewing sarcoma [10], [11]. We also included the third top hit, RBM14, for the validation of their binding with JQ1. V5-fused proteins were purified and incubated with Bio-JQ1 or Bio immobilized on streptavidin beads. V5-fused bromodomains (BD1 and BD2) from BRD4 were also purified and used as the positive controls. The protein pull-down assays showed that EWSR1 strongly associates with Bio-JQ1, while the binding of EYA3 with Bio-JQ1 was weak (Figure 3B). However, we couldn’t identify the interaction of RBM14 with Bio-JQ1.

Given that JQ1 demonstrates the excellent shape complementarity with the acetyl-lysine binding cavity [12], we next determined whether EWSR1 is capable of binding to acetylated histones. The biotinylated histone H4 proteins, either acetylated (Bio-H4ac) or unacetylated (Bio-H4), were immobilized on streptavidin beads, which were further incubated with V5-fused EWSR1 protein. *In vitro* protein pull-down assays showed that EWSR1 preferentially binds to Bio-H4ac (Figure 3C). The interaction between V5-tagged EWSR1 and acetylated H4 histone was also confirmed *in vivo* (Figure 3D). Such interaction can be efficiently blocked by JQ1 (Figure 3E), confirming that EWSR1 also recognizes the acetylated histones and could be an alternative protein target of JQ1.

### PLATO-BC for identifying peptide-protein interaction

A short linear peptide is able to associate with a globular protein receptor and form modular binding motif [13]. Peptide-protein interactions play key functional roles in the living cells, and account for a significant portion (15%–40%) of overall protein–protein interactions [14]. We test whether PLATO-BC is capable of identifying protein targets of small peptides. Ubiquitin acts as a signaling peptide that regulates a wide range of cellular processes [15]. Ubiquitin signals are recognized by ubiquitin-binding proteins (ubiquitin receptors), which transmit signals to the downstream biological cascades in cells.

**Figure 4:**
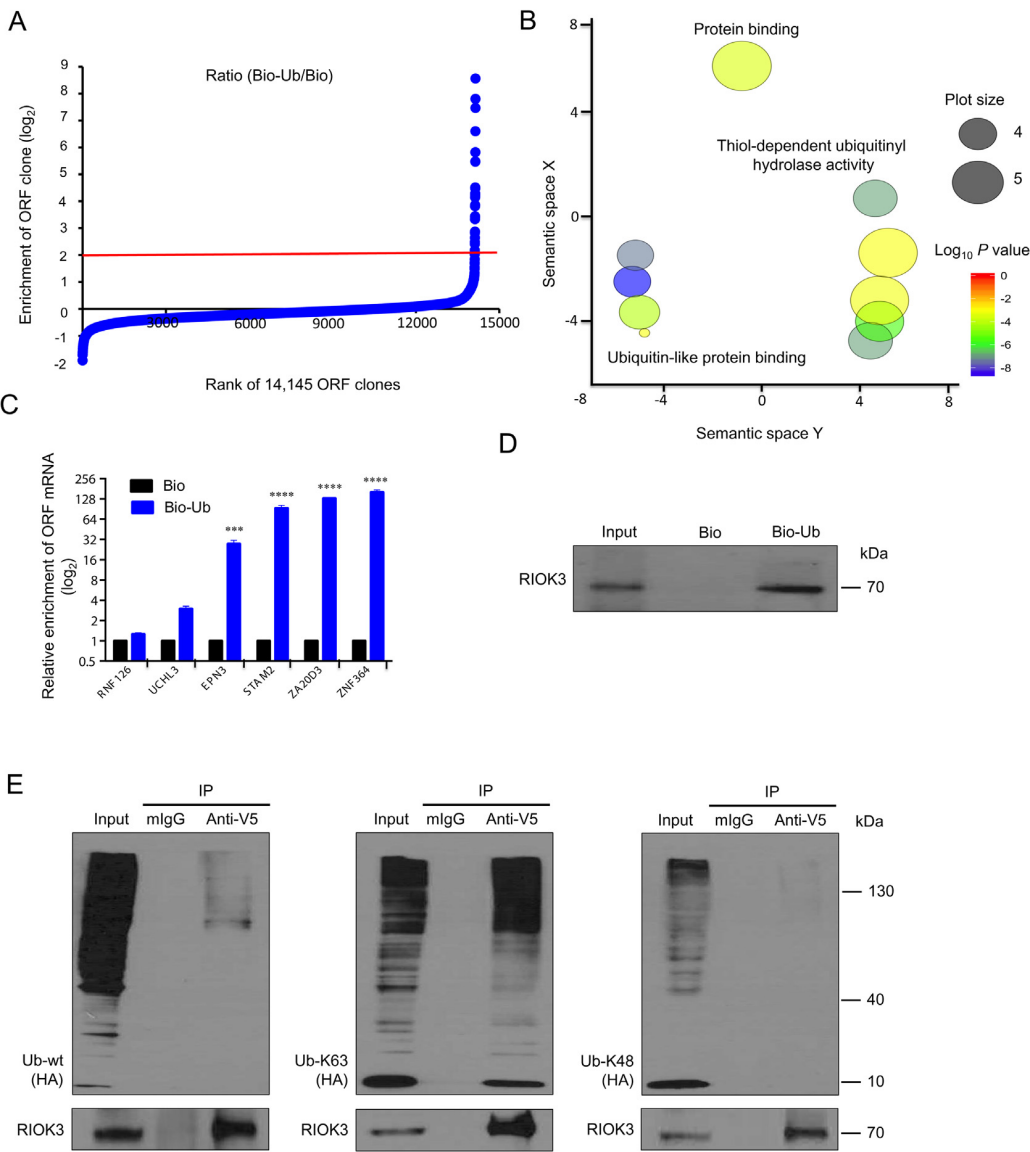
**Identification of protein binders for the free ubiquitin peptide by using PLATO-BC assay** **A.** Rank of ORF enrichment from the PLATO-BC assays for biotinylated ubiquitin (Bio-Ub) relative to free biotin (Bio). 14,145 ORFs were ranked according to the ratio of Bio-Ub/Bio (from the smallest to the largest). The red line shows the cut-off value. **B.** GO analysis of top-ranked ORFs was performed using GOrilla. Clustering analysis and visualization were performed using REVIGO. Circle size represents the uniqueness of the GO term in EBI GOA database (more general terms have larger circle size). Circle color indicates the *P* value. **C.** mRNA enrichment of a set of known ubiquitin-binding proteins from the PLATO-BC assay of Bio-Ub. mRNA level was quantified by RT-qPCR. Relative enrichment of ubiquitin-binding proteins was normalized to Bio. Data are presented as mean ± SD (*n* = 3). ***, *P <* 0.001; ****, *P <* 0.0001, ANOVA. **D.** Interaction of RIOK3 with ubiquitin *in vitro*. pET-DEST42-RIOK3 was transformed into the *E. coli* DE3 strain. V5-His6-tagged RIOK3 protein was purified and subjected to protein pull-down assays for Bio-Ub or Bio. Protein samples were separated by SDS-PAGE and analyzed by immunoblotting using an anti-V5 antibody. **E.** The preferable interaction of RIOK3 with K63-polyubiquitin chains. pcDNA-DEST43 was co-transfected into HEK293T cells with the vector expressing HA-tagged wild-type (wt), K63- or K48-specific ubiquitin. Cell lysate was prepared and subjected to the protein immuno-precipitation assays for V5-His6-tagged RIOK3 using an anti-V5 antibody or a mouse IgG (mIgG) control. Protein samples were separated by SDS-PAGE and analyzed by immunoblotting using an anti-HA antibody. GO, gene ontology; RIOK3, RIO kinase 3; Ub, ubiquitin.

We used PLATO-BC to identify previously undiscovered proteins that bind to the ubiquitin peptide. We immobilized the N-terminal biotinylated ubiquitin (Bio-Ub) or free biotin (Bio) on streptavidin magnetic beads, which was subjected to PLATO-BC analysis. The ORF clones were ranked according to the ratio of enrichment (Bio-Ub/Bio) (Figure 4A). We identified 29 top-ranked protein candidates (cutoff, log_2_ = 2), and the GO analysis using GOrilla revealed that this list of ORFs is highly enriched with Ub-binding proteins (Figure 4B, Table S1). Using qPCR, we validated that the PLATO-BC method results in the Bio-Ub enrichment of several Ub-binding proteins, including ubiquitin C-terminal hydrolase 3 (UCHL3), epsin 3 (EPN3), signal transducing adaptor molecule 2 (STAM2), zinc finger AN1-type containing 6 (ZA20D3), and RNF115 ring finger protein 115 (ZNF364) (Figure 4C). We also identified a previously unreported Ub-binding protein, right open reading frame kinase 3 (RIOK3). RIOK3 is a serine/threonine kinase belonging to the RIO kinase gene family. RIOK3 is involved in the antiviral signaling pathways by phosphorylating IFIH1 interferon induced with helicase C domain 1 (MDA5) [16] as well as by bridging the interaction between TNAK binding kinase 1 (TBK1) and interferon regulator factor 3 (IRF3) [17]. V5-fused RIOK3 protein was purified and incubated with Bio-Ub or Bio immobilized on streptavidin beads. *In vitro* protein pull-down assays showed that RIOK3 strongly associates with Bio-Ub (Figure 4D).

Ub is often covalently conjugated to substrates as the polymer and poly-Ub chains can be formed on the defined lysine residues of Ub. K48- and K63-linked Ub chains are the two most abundant chain types, which regulate proteolytic and non-proteolytic functions, respectively [18]. We cloned the RIOK3 ORF into the pcDNA-DEST40 vector, and co-transfected it into HEK293T cells, with the HA-tagged wild-type Ub (HA-Ub-wt), HA-Ub-K48, or HA-Ub-K63, in which all lysine residues of Ub were mutated to arginine except the K48 or K63, respectively. We found that V5-tagged RIOK3 protein was immunoprecipitated from cell lysates, and only K63-linked, not K48-linked, Ub chains associated and co-precipitated with V5-RIOK3 (Figure 4E), indicating that RIOK3 is Ub-binding protein that specifically recognizes K63-linked Ub chains.

### PLATO-BC for identifying protein–protein interaction

The vast majority of proteins interact with other proteins. The function of a protein is usually context-dependent and often modulated by other proteins with which it interacts. This is particularly true for host-virus interactions, since viruses generally encode a limited number of proteins that interact with various host proteins and play multiple roles in viral replication and pathogenesis [19]. ZIKV, a single-stranded RNA virus of the Flaviviridae family, has recently been linked to unexpected upsurge in the infants born with microcephaly and become a global health threat [20]. NS5 is essential for the replication of viral RNA genome, due to its N-terminal methyltransferase (MTase) and C-terminal RNA-dependent RNA polymerase (RdRp) activities [21]. NS5 protein is also known to suppress type I interferon (IFN) response [22], [23] but enhance the activity of type II IFN [24].

**Figure 5:**
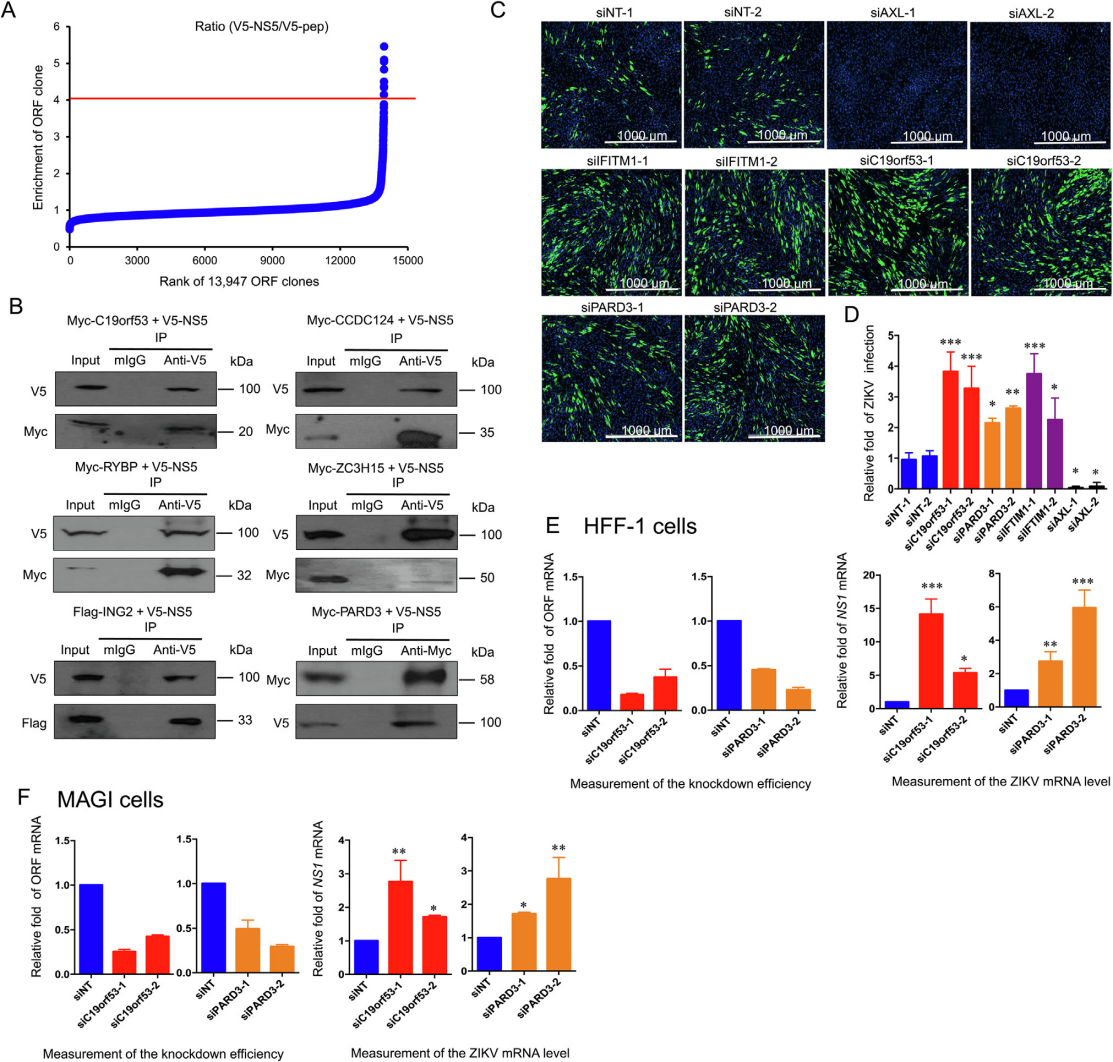
**Identification of host protein binders for the ZIKV-NS5 protein by using PLATO-BC assay** **A.** Rank of ORF enrichment from the PLATO-BC assays for V5-His6-tagged ZIKV-NS5 protein (V5-NS5) relative to a short peptide (V5-pep). 13,947 ORFs were ranked according to the ratio of V5-NS5/V5-pep (from the smallest to the largest). The red line shows the cut-off value. pcDNA-DEST40 expressing V5-tagged ZIKV-NS5 was transiently transfected in HEK293T cells. Cell lysate was prepared and subjected to the protein immunoprecipitation assays for V5-NS5 using an anti-V5 antibody. The precipitated protein samples were subjected to the PLATO-BC assays. **B.** Validation of host proteins interacting with ZIKV-NS5 using protein co-immunoprecipitation assays. pcDNA-DEST40-ZIKV-NS5 was co-transfected into HEK293T cells with the pEZY vector expressing Myc or FLAG tagged protein candidates. For majority of validations, cell lysate was prepared and subjected to the protein immuno-precipitation assays for V5-His6-tagged ZIKV-NS5 using an anti-V5 antibody or a mouse IgG (mIgG) control. Protein samples were separated by SDS-PAGE and analyzed by immunoblotting using anti-V5, and anti-Myc, or FLAG antibody. For PARD3 validation, an anti-Myc antibody or a mIgG control was used for immunoprecipitation. **C.** Knockdown of *C19orf53* and *PARD3* by RNAi increases ZIKV infection. HFF-1 cells were transiently transfected with indicated siRNAs, and then infected with ZIKV. Cells were stained for expression of ZIKV capsid protein. Fluorescent images of ZIKV capsid (green) and nuclei (blue) are illustrated for HFF-1 cells treated with non-targeting siRNAs (siNTs), or siRNAs targeting known ZIKV restriction (*IFTM3*) and dependency (*AXL*) factors, as well as *PARD3* and *C19orf53*. Two unique siRNAs were used for each gene. **D.** The infection efficiency is measured by calculating the ratio (green cells/nucleus). Relative fold of ZIKV infection in HFF-1 cells transfected with siRNAs above was normalized to those with siNTs. **E.** Total mRNAs were extracted from siRNA-transfected HFF-1 cells, and subjected to reverse transcription and qPCR to measure knockdown efficiency of siRNA and relative *NS1* mRNA level. Relative mRNA level of *C19orf53* or *PARD3* siRNA knockdown was normalized to that of siNT in HFF-1 cells. Relative mRNA level of ZIKV NS1 was measured in HFF −1 cells with knockdown of *C19orf53* or *PARD3*. **F.** Total mRNAs were extracted from siRNA-transfected MAGI cells, and subjected to reverse transcription and qPCR to measure knockdown efficiency of siRNA and relative *NS1* mRNA level. Relative mRNA level of *C19orf53* or *PARD3* siRNA knockdown was normalized to that of siNT in MAGI cells. Relative mRNA level of ZIKV NS1 was measured in MAGI cells with knockdown of *C19orf53* or *PARD3*. Data are presented as mean ± SD (*n* = 3). *, P < 0.05; **, P < 0.01; ***, P < 0.001, ANOVA. ZIKV, Zika virus; NS5, nonstructural protein 5; PARD3, par-3 family cell polarity regulator; C19orf53, chromosome 19 open reading frame 53; IFITM3, interferon-induced transmembrane protein 3; AXL, AXL receptor tyrosine kinase; NS1, nonstructural protein 1.

We used PLATO-BC to identify previously undiscovered proteins that interact with a ZIKV-encoded protein NS5. We cloned the NS5 ORF or a flag peptide, into the pcDNA-DEST40 vector. V5-tagged NS5 (V5-NS5) or peptide (V5-pep) was immunoprecipitated using the anti-V5-tag mAb magnetic beads in 293T cells, which was subjected to PLATO-BC analysis. The ORF clones were ranked according to the ratio of enrichment (V5-NS5/V5-pep) (Figure 5A). We identified 7 top-ranked protein candidates (cutoff = 4), which preferentially bind to V5-NS5 (Table S1). To validate these protein interactions, we cloned the host ORFs including *PARD3*, *C19orf53*, *CCDC124*, *RYBP*, C11orf 46 *ZC3H15*, and *ING2* into the pEZY vector and co-transfected them individually with pcDNA-DEST40 vector expressing V5-NS5 into HEK293T cells. The cloning of C11orf46 ORF was unfortunately unsuccessful. Through the co-immunoprecipitation of V5-NS5, we identified that all six of Myc-tagged or Flag-tagged host proteins (C19orf53, CCDC124, RYBP, ZC3H15, ING2, and PARD3) associated and co-precipitated with V5-NS5 (Figure 5B).

We next determined whether these host proteins might functionally regulate ZIKV viral replication. We transfected the gene-specific siRNAs into the HFF-1 cells, followed by the inoculation of ZIKV. We also included the known ZIKV replication inhibiting and supporting genes, *IFITM3* and *AXL*, respectively, for these experiments. We showed that knockdown of *C19orf53* and *PARD3* modestly increased ZIKV viral replication, which was comparable to the effect of *IFITM3* knockdown (Figure 5C and D). However, knockdown of other host proteins failed to produce any significant effect (Figure S1).

The knockdown efficiency of C19orf53 and PARD3 mediated by siRNA was measured using the qPCR analysis. The NS1 mRNA of ZIKV increased when *C19orf53* or *PARD3* was knockdown in HFF-1 cells (Figure 5E). The effect of *C19orf53* or *PARD3* on ZIKV replication was also tested in MAGI cells. Knockdown of *C19orf53* or *PARD3* in MAGI cells significantly enhanced ZIKV replication (Figure 5F). These results suggest that the PLATO-BC enables the identification of novel host proteins that interact with ZIKV NS5 protein, and these host proteins may interfere with ZIKV viral replication.

## Discussion

In this report, we demonstrated the expanded applications of PLATO-BC, which was mainly improved by barcoding each ORF clone to simplify the sample preparations in previous work. Our results showed that PLATO-BC was successful to identify novel protein interactions for various baits, including antisera, small-molecule compounds, peptides, and proteins.

We have tested the PLATO-BC system on the same set of antisera samples from IBM patients that were subjected to antigen identification using PLATO. From these earlier studies, NT5C1A was identified as a potential autoantigen that associates with IBM [5]. The use of PLATO-BC system still faithfully identified NT5C1A as a top-ranked IBM autoantigen (Figure 2A and B). Furthermore, PLATO-BC analysis also identified TRIM21 as another potential IBM autoantigen, suggesting that PLATO-BC could be more sensitive for protein interaction discovery. Members of TRIM protein family usually encode E3 ubiquitin ligase and have been proposed to associate with many autoimmune conditions [25], [26]. The previous studies using PLATO have identified a set of TRIM proteins (TRIM1/MID2, TRIM18/MID1, TRIM54 and TRIM55) as cancer autoantigens of paraneoplastic neurological disorder (PND) [4], [5]. TRIM21 was previously identified as an autoantigen for several autoimmune conditions, including Sjögren syndrome [27], [28], rheumatoid synovitis [29], systemic sclerosis[30], and systemic lupus erythematosus [31]. TRIM21-specific antibodies thus hold the great potential as a biomarker for these autoimmune conditions [32]. TRIM21 negatively regulates multiple immune signals by targeting protein substrates to proteasome degradation [33], which may explain why TRIM21 highly associates with autoimmune conditions, including IBM.

Using a biotinylated BETi, JQ1, as an example, we also demonstrated that PLATO-BC enables the discovery of novel protein targets for small-molecule compounds. JQ1 preferentially binds to the bromodomain-containing proteins, and there were no previous reports showing that JQ1 may also interact with other types of proteins. However, our PLATO-BC analysis identified that EWSR1 and EYA3 can also be the protein targets of JQ1. Surprisingly, these two top-ranked hits are both involved in the Ewing’s sarcoma, a rare bone and soft tissue tumor that mostly occurs in children and young adults [11]. EWSR1 fuses with an E26 transformation specific (ETS) transcription factor, usually FLI1, to form the EWSR1-FLI1 oncogene that promotes Ewing’s sarcoma. EYA3, a DNA repair protein and transcriptional cofactor, is highly expressed in Ewing’s sarcoma cells and required for their survival and chemoresistance [11]. Particularly, our results identified that EWSR1 protein strongly binds to JQ1 (Figure 3B). We further showed that EWSR1 associates with acetylated histone H4 and JQ1 inhibits such interaction (Figure 3C–E), suggesting that EWSR1 may act as an epigenetic reader that directly recognizes histone acetylation signals. Although EWSR1 contains no bromodomains, we couldn’t rule out the possibility that EWSR1 protein may contain other un-characterized domain(s) that may mimic bromodomains and also recognize acetylated histones. Solving the crystal structure of EWSR1 protein may help to prove this hypothesis. Nonetheless, other studies indeed show that JQ1 suppresses the EWSR1-FLI1-driven gene transcription in Ewing’s sarcoma cells [34], [35], and that the EWSR1-FLI1-bound chromatins correlate with H3K27 acetylation [36]. These results seem to support that EWSR1 may be a novel histone acetylation reader targeted by JQ1.

We evaluated the capability of PLATO-BC to identify protein receptors for small peptides, such as ubiquitin. Our results are encouraging, since most of top-ranked hits, 24 out of 29 (83%), identified from PLATO-BC assay, are known ubiquitin-binding proteins (Figure 4B and C). We expect that PLATO-BC should be useful to identify protein receptors for other peptides as well, such as SUMO and ISG15. RIOK3, one of top hits from PLATO-BC assay, was not previously reported as a ubiquitin-binding protein. We were able to use the alternative protein pull-down assay to confirm that RIOK3 binds to both free ubiquitin and polyubiquitin chains, preferentially K63-linked ones (Figure 4D and E). RIOK3 is a cytoplasmic protein kinase that both positively and negatively regulates type I interferon production as well as interferes with NF-kB signaling pathway [17], [37]. K63-linked ubiquitination is increasingly important in immune signaling, and many players, particularly in NF-kB and RIG-I pathways, are protein substrates of K63-linked ubiquitination that affects signaling transduction more than protein degradation [38]. We postulate that RIOK3 may regulate the activity of certain protein components in immune signaling by interacting with their K63-linked ubiquitin chains.

Lastly, we demonstrated that PLATO-BC could be applied for discovery of protein–protein interactions. We identified novel host proteins that interact with a viral protein, NS5 protein from ZIKV. ZIKV-NS5 is a large (∼103 KDa) non-structural protein that possesses the N-terminal MTase domain for viral RNA capping and the C-terminal RdRp domain for viral RNA synthesis [21], [39]. Surprisingly, although ZIKV RNA replication, mediated by NS5, is believed to be exclusively cytoplasmic, a significant portion of ZIKV-NS5 resides in the nucleus of the infected cells, where it is thought to play a role in modulation of the host antiviral response [40], [41], [42]. ZIKV-NS5 has recently been shown to suppress type I IFN signaling by targeting STAT2 for degradation [22], [23] but activate type II IFN signaling by promoting the formation of STAT1-STAT1 protein complexes [24]. These studies indicate that ZIKV-NS5 associates with a variety of host proteins to elicit multiple functions in ZIKV viral replication. We were able to identify a set of previously unreported host proteins that bind to ZIKV-NS5 from the PLATO-BC analysis (Figure 5A), and validate these interactions using the traditional protein pull-down assay (Figure 5B). However, such interactions remain to be validated using virus-derived NS5 protein in cells infected with ZIKV, so that the microenvironment for NS5 interaction with host proteins, including the presence of viral RNAs and other nonstructural proteins, will be included for consideration. We further showed that two NS5-binding host proteins, PARD3 and C19orf53 (LYPG10), might negatively regulate ZIKV viral replication (Figure 5C and D). PARD3 is cell polarity regulator that is involved not only in cell proliferation and apoptosis [43] but also in regulation of STAT3 signaling [44], [45]. The cellular function of C19orf53 (LYPG10) is unknown, but it is indeed a nucleolar protein. We will need to further characterize these NS5-binding proteins in terms of their impact on NS5-mediated viral RNA synthesis, antiviral signaling modulation, or other un-recognized activities. We used AXL protein as a positive control since AXL is known to support ZIKV infection [46]. AXL is a receptor tyrosine kinase and is postulated as a receptor for mediating ZIKV entry in several cell lines [46], although a recent study has also shown that AXL is not an indispensable factor for ZIKV infection in mice [47]. Our studies to identify new host proteins interacting with ZIKV viral proteins will improve the understanding of ZIKV-host interactions and facilitate the development of novel antiviral strategies.

PLATO-BC is improved for the identification of new protein interactions. We identified the known IBM autoantigen, NT5C1A, which is a top-ranked hit (Figure 2A and B), although it was from some but not all tested patient samples – probably due to the heterogeneity of immune responses across patients. In addition, BRD2, a known JQ1-binding protein, was also identified as a top hit (Figure 3A). The more striking result is that among the top-ranked protein hits from PLATO-BC assay for free ubiquitin peptide, most are known ubiquitin-binding proteins (24 out of 29, 83%), which undoubtedly validates the use of PLATO-BC for protein interaction discovery. PLATO-BC also dramatically facilitates the recognition of ORF identities. Instead of the tedious process to recover the unique 3′ end of each ORF for DNA sequencing, now we can pair barcode and ORF information and only recover barcode identities for ORF de-convolution with much simplified steps (Figure 1). In addition, numerous steps for 3′ end recovery may cause the loss of certain ORFs during sample preparation, which could be avoided by barcode recovery. However, we did notice that certain top-ranked hits from PLATO-BC assay, including the RBM14, couldn’t be validated by alternative approaches. One possibility is that certain *in vitro* ribosome-displayed ORFs may lack proper protein folding and/or protein post-translational modifications, resulting in the potential false-positive discovery. Nevertheless, we were still able to verify the majority of top hits across all tested baits, justifying the use of PLATO-BC for protein interaction discovery. Another issue is that our current ORFeome collections are still not completed, contributing to the false-negative discovery, although completion of entire human ORFeome is still keeping improved over the time. PLATO-BC exhibits certain advantages over several traditional methods. For example, it is relatively hard to display long polypeptides on phage, bacteria, or yeast. These methods also suffered from highly skewed clonal abundance. In addition, these systems generally cannot be used for antibody analysis or protein target identification of compounds. Although protein microarrays are quite useful for identifying protein interactions, individual proteins would need to be purified and immobilized, which is labor intensive and quite costly. Therefore, although PLATO-BC bears several limitations, it will still be a useful method to expand the proteomic toolkits for protein interaction discovery.

## Conclusion

In summary, our newly developed PLATO-BC method significantly simplifies the sample preparation and improves its application for protein interaction discovery. As a proof of principle, we used the PLATO-BC method to successfully identify the autoantigens of antisera, the protein targets of compounds, as well as the protein binders of short peptides or full-length proteins. These newly-recognized protein interactions were validated using alternative approaches. The much simpler sample preparations will allow the use of PLATO-BC for massive protein interaction projects to screen large numbers of samples, such as cohort-scale autoantibody profiling and structure–activity relationship analyses of small-molecule compounds. However, several limitations still associate with PLATO-BC and require further improvement [4]. For example, there is still lack of cellular machineries in *in vitro* PLATO-BC system to support proper protein folding and post-translational modifications. These components will be included for testing to further improve PLATO-BC in future. Nevertheless, PLATO-BC is a valuable platform that can complement with currently available proteomic tools for versatile protein interaction discoveries.

## Authors’ contributions

JZ and SE conceived the study. WK performed cell culture, RT-qPCR, Pull-down, Western blot assay. WK and TH performed siRNA reverse transfection assay. WK, NS, and JZ performed the sequencing and the following analyses. WK and JZ drafted the manuscript. JZ provided overall supervision of the study. GF, QX, ML, JQ, SL, WZ, and JQ contributed to the discussion. All author had read and approved the final manuscript.

## Competing interests

The authors have declared no competing interests.

## Supplementary data

Supplementary Figure S1 **Knockdown of other hits did not affect ZIKV replication** HFF-1 cells were transiently transfected with indicated siRNAs, and then infected with ZIKV. Relative fold of ZIKV infection in HFF-1 cells transfected with different siRNAs was normalized to those with siNTs.
