# Supplementary figures and images for: Diversified Application of Barcoded PLATO (PLATO-BC) Platform for Identification of Protein Interactions
Source: Genomics Proteomics Bioinformatics. 2019 Sep 5;17(3):319–31. doi: 10.1016/j.gpb.2018.12.010 (PMC6818353; doi:10.1016/j.gpb.2018.12.010)

## Slide 1
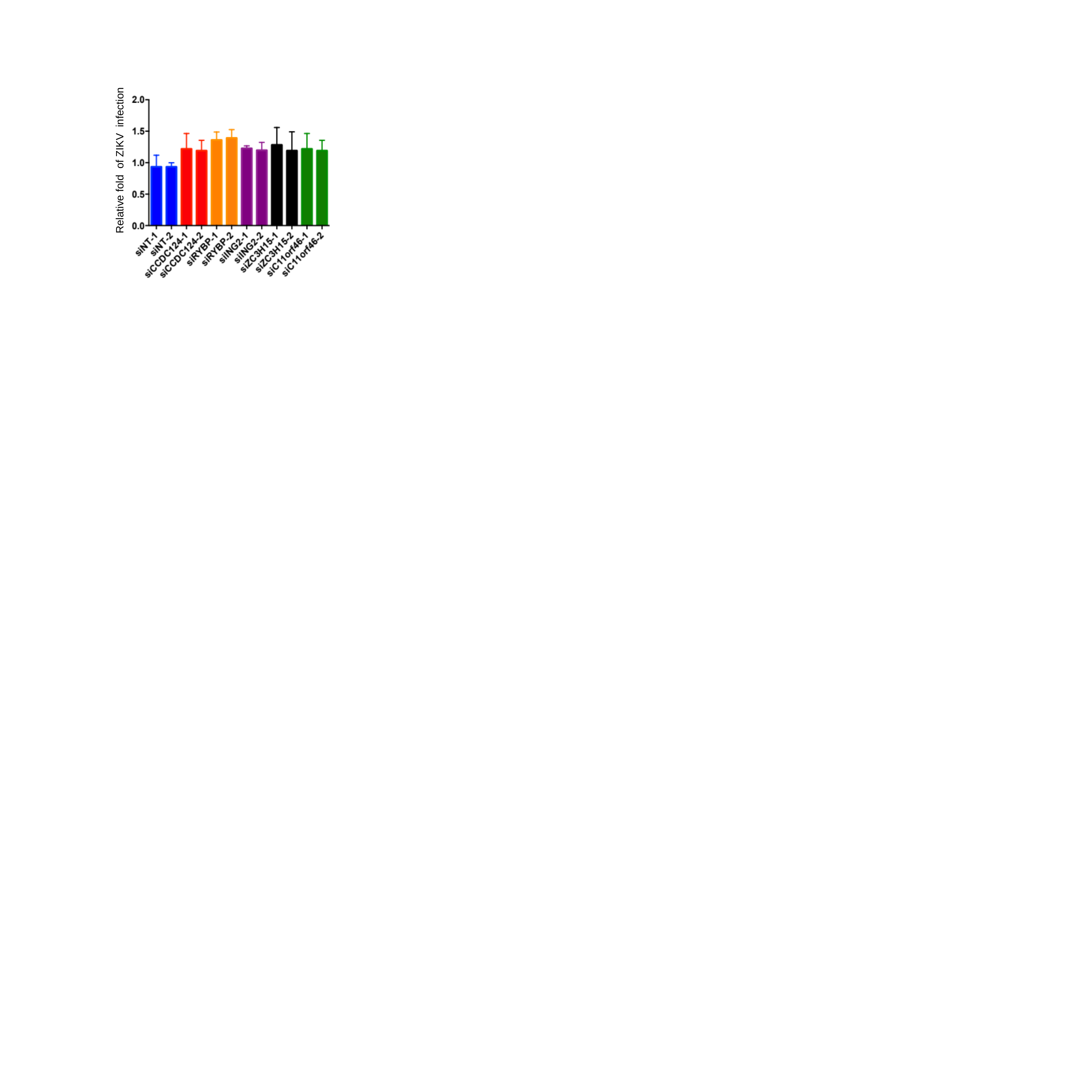

Relative fold of ZIKV infection

Supplement: Supplementary Figure S1 — Knockdown of other hits did not affect ZIKV replication HFF-1 cells were transiently transfected with indicated siRNAs, and then infected with ZIKV. Relative fold of ZIKV infection in HFF-1 cells transfected with different siRNAs was normalized to those with siNTs. [file mmc1.pptx]
