# Supplementary material for: Diversified Application of Barcoded PLATO (PLATO-BC) Platform for Identification of Protein Interactions
Source: Genomics Proteomics Bioinformatics. 2019 Sep 5;17(3):319–31. doi: 10.1016/j.gpb.2018.12.010 (PMC6818353; doi:10.1016/j.gpb.2018.12.010)
Supplement: Supplementary Table S1 [file mmc2.docx]

| **Gene name** | **Catalog No.** | **siRNA sequence** |
| --- | --- | --- |
| *AXL* | s1846 | GGGUGGAGGUUAUCCUGAATT |
| *AXL* | s1847 | CAGCGAGAUUUAUGACUAUTT |
| *CCDC124* | s41756 | CCGCGGCUCAAACAAGAGATT |
| *CCDC124* | s225505 | ACAAACACGUCAUGAGGAATT |
| *C11orf46* | s42387 | GGUUCAGACAGACAAGUGATT |
| *C11orf46* | s42388 | ACUUAGAACUGGAAUGACATT |
| *C11orf53* | s26334 | GGAAGAAGAUCGAACAUGATT |
| *C11orf53* | s226241 | CGCAAAGAGUAAGACGGCATT |
| *IFITM3* | s195034 | CAGGCCUAUGGAUAGAUCAT |
| *IFITM3* | s195035 | CCCACGUACUCCAACUUCCTT |
| *ING2* | s7431 | GAUAGGAUGUGACAAUGAATT |
| *ING2* | s7433 | GUGUUUCACUUACCUAUAATT |
| *PARD3* | s32126 | GGAUGAUAGAGAACGAAGATT |
| *PARD3* | s32128 | GGAUUUCGUUAAAACACGATT |
| *RYBP* | s23812 | GAAACAGUGCUGAAGCCUUTT |
| *RYBP* | s23813 | CAAAGACCAGCGAAACAAATT |
| *ZC3H15* | s31666 | GUCCUGAACUGGUCAAUGATT |
| *ZC3H15* | s31667 | GGAGAUAAGUGUAAGUUCUTT |

**Table S1 List of siRNAs used in this study**

*Note:* All siRNAs were purchased from Ambion, Austin, TX.
