# Supplementary material for: Diversified Application of Barcoded PLATO (PLATO-BC) Platform for Identification of Protein Interactions
Source: Genomics Proteomics Bioinformatics. 2019 Sep 5;17(3):319–31. doi: 10.1016/j.gpb.2018.12.010 (PMC6818353; doi:10.1016/j.gpb.2018.12.010)
Supplement: Supplementary Table S2 [file mmc3.docx]

| **Gene name** | **Forward primer (5′–3′)** | **Reverse primer (5′–3′)** |
| --- | --- | --- |
| *RNF126* | CAGTTTGCTTTCGGCATCTTC | CGTAGTCCATAGGGTTTGAGTG |
| *ZA20D3* | AGAAGCCCAGTCAGCATTAG | TTTCCACACCGGCATTCA |
| *ZNF364* | CCTGACAGATCTCCAGCTATTG | CGAGTAGAGTCCTCACCATTTAAG |
| *TAM2* | CCCATCAAGGCATAGGGTTATT | AAAGGGCCTCCATCACTTTC |
| *EPN3* | CCTCCTACAACTCCTCCTCTT | CTCCTCCTCCTTTCTCTCTTCT |
| *UCHL3* | GAGGTCACCAACCAGTTTCTTA | GGCATCATAGTTCTCCAGGTATC |
| *BC-Input* | GTATATTCGCCGCACCGTAGT | CTGCTTCGGCCTTAGCTTTCG |
| *GAPDH* | GGGTGTGAACCATGAGAAGT | GTAGAGGCAGGGATGATGTT |
| *C19orf53* | CCAGCAATGACCAGGAAGATAC | TGGGCACATGGTTGCTAAA |
| *PARD3* | GGAACATGGAGATGGAGGAATAC | GAAGTGGAGAGGCCAATTAGAG |
| *ZIKV NS1* | TGGAGTTCAACTGACGGTCG | TACCCCGAACCCATGATCCT |

**Table S2 List of qPCR primers used in this study**
