# Supplementary material for: Diversified Application of Barcoded PLATO (PLATO-BC) Platform for Identification of Protein Interactions
Source: Genomics Proteomics Bioinformatics. 2019 Sep 5;17(3):319–31. doi: 10.1016/j.gpb.2018.12.010 (PMC6818353; doi:10.1016/j.gpb.2018.12.010)
Supplement: Supplementary Table S3 [file mmc4.docx]

**Table S3 Hit list of the PLATO-BC assays for JQ1**

| **Gene ID** | **Gene name** | **Ratio (Biotin-JQ1/Biotin)** | **Gene ID** | **Gene name** | **Ratio (Biotin-JQ1/Biotin)** |
| --- | --- | --- | --- | --- | --- |
| 2130 | *EWSR1* | 9.91 | 7782 | *SLC30A4* | 4.95 |
| 2140 | *EYA3* | 8.42 | 79781 | *IQCA* | 4.94 |
| 10432 | *RBM14* | 6.81 | 2631 | *GBAS* | 4.93 |
| 965 | *CD58* | 6.40 | 23568 | *ARL2BP* | 4.90 |
| 6046 | *BRD2* | 6.33 | 2191 | *SIMP* | 4.90 |
| 54959 | *APIN* | 5.93 | 11056 | *DDX52* | 4.89 |
| 2774 | *GNAL* | 5.64 | 84135 | *FLJ12787* | 4.83 |
| 7103 | *TSPAN8* | 5.54 | 10597 | *SEDLP* | 4.83 |
| 5520 | *PPP2R2A* | 5.49 | 9447 | *AIM2* | 4.82 |
| 9814 | *SFI1* | 5.44 | 55161 | *TMEM33* | 4.82 |
| 11014 | *KDELR2* | 5.40 | 7730 | *ZNF177* | 4.78 |
| 11014 | *KDELR2* | 5.33 | 57669 | *EPB41L5* | 4.72 |
| 84791 | *MGC14801* | 5.28 | 1368 | *CPM* | 4.70 |
| 51227 | *DSCR5* | 5.20 | 286148 | *LOC286148* | 4.62 |
| 9647 | *SH3BP5* | 5.19 | 10350 | *ABCA9* | 4.59 |
| 84140 | *FLJ13305* | 5.18 | 6747 | *SSR3* | 4.57 |
| 79669 | *TTMP* | 5.18 | 55028 | *HLC-8* | 4.56 |
| 51174 | *TUBD1* | 5.15 | 11153 | *HYPE* | 4.55 |
| 84294 | *MGC14595* | 5.15 | 116441 | *TM4SF18* | 4.55 |
| 51602 | *NOP5/NOP58* | 5.11 | 120534 | *FLJ38968* | 4.50 |
| 374879 | *FLJ38144* | 5.06 | 7639 | *ZNF85* | 4.50 |
| 120103 | *SLC36A4* | 5.06 | 2623 | *GATA1* | 4.49 |
| 11160 | *SPFH2* | 4.99 | 6309 | *SC5DL* | 4.49 |
| 8477 | *GPR65* | 4.98 | 11126 | *CD160* | 4.49 |
| 23390 | *ZDHHC17* | 4.98 | 93624 | *MGC21874* | 4.45 |
| 84935 | *FLJ14834* | 4.96 | 219749 | *ZNF25* | 4.45 |
| 81899 | *FAHD1* | 4.44 | 54619 | *CCNJ* | 4.23 |
| 8535 | *CBX4* | 4.44 | 1069 | *CETN2* | 4.22 |
| 55076 | *TMEM45A* | 4.43 | 55023 | *PHIP* | 4.21 |
| 4507 | *MTAP* | 4.43 | 10878 | *CFHL3* | 4.21 |
| 27179 | *GPR82* | 4.41 | 25819 | *CCRN4L* | 4.20 |
| 9698 | *PUM1* | 4.39 | 23151 | *KIAA0767* | 4.20 |
| 7430 | *VIL2* | 4.39 | 84618 | *NT5C1A* | 4.19 |
| 771 | *CA12* | 4.38 | 84897 | *TBRG1* | 4.19 |
| 55023 | *PHIP* | 4.38 | 2966 | *GTF2H2* | 4.17 |
| 7711 | *ZNF155* | 4.38 | 27123 | *DKK2* | 4.15 |
| 54504 | *CPVL* | 4.38 | 219437 | *OR5L1* | 4.14 |
| 55151 | *TMEM38B* | 4.35 | 26051 | *PPP1R16B* | 4.14 |
| 6555 | *SLC10A2* | 4.35 | 241 | *ALOX5AP* | 4.14 |
| 7690 | *ZNF131* | 4.35 | 7768 | *ZNF225* | 4.14 |
| 8780 | *RIOK3* | 4.35 | 1672 | *DEFB1* | 4.13 |
| 8395 | *PIP5K1B* | 4.33 | 1615 | *DARS* | 4.13 |
| 84924 | *ZNF566* | 4.33 | 5906 | *RAP1A* | 4.13 |
| 26248 | *OR2K2* | 4.32 | 132228 | *FLJ38608* | 4.13 |
| 3458 | *IFNG* | 4.32 | 10562 | *OLFM4* | 4.13 |
| 55319 | *FLJ11184* | 4.31 | 54555 | *DDX49* | 4.12 |
| 10096 | *ACTR3* | 4.31 | 10874 | *NMU* | 4.12 |
| 9108 | *MTMR7* | 4.29 | 5191 | *PEX7* | 4.11 |
| 8036 | *SHOC2* | 4.28 | 57522 | *SRGAP1* | 4.10 |
| 5597 | *RGS2* | 4.27 | 51177 | *CKIP-1* | 4.10 |
| 2706 | *GJB2* | 4.26 | 10629 | *TAF6L* | 4.10 |
| 6166 | *RPL36AL* | 4.26 | 8514 | *KCNAB2* | 4.09 |
| 79887 | *FLJ22662* | 4.25 | 51633 | *CGI-77* | 4.04 |
| 167227 | *DCP2* | 4.07 | 7597 | *ZNF46* | 4.02 |
| 340156 | *LOC340156* | 4.07 | 51026 | *GOLT1B* | 4.02 |
| 50943 | *FOXP3* | 4.06 | 143686 | *SESN3* | 4.01 |
| 26253 | *CLEC4E* | 4.06 | 196477 | *C12orf12* | 4.01 |
| 9331 | *B4GALT6* | 4.06 | 150159 | *LOC150159* | 4.01 |
| 2969 | *SPIN* | 4.06 | 57180 | *ARP3BETA* | 4.01 |
| 131831 | *MGC39662* | 4.06 | 1814 | *DRD3* | 4.00 |
| 200728 | *TMEM17* | 4.05 |  |  |  |
