# Supplementary material for: Diversified Application of Barcoded PLATO (PLATO-BC) Platform for Identification of Protein Interactions
Source: Genomics Proteomics Bioinformatics. 2019 Sep 5;17(3):319–31. doi: 10.1016/j.gpb.2018.12.010 (PMC6818353; doi:10.1016/j.gpb.2018.12.010)
Supplement: Supplementary Table S4 [file mmc5.docx]

**Table S4 Hit list of the PLATO-BC assays for ubiquitin**

| **Gene ID** | **Gene name** | **Ratio (Biotin-Ub/Biotin)** | **Gene ID** | **Gene name** | **Ratio (Biotin-Ub/Biotin)** |
| --- | --- | --- | --- | --- | --- |
| 54469 | *ZA20D3* | 377.48 | 119594473 | *LOC51035* | 14.09 |
| 10043 | *TOM1* | 223.55 | 55658 | *RNF126* | 10.85 |
| 27246 | *ZNF364* | 176.96 | 55658 | *RNF126* | 10.60 |
| 7345 | *UCHL1* | 97.19 | 7347 | *UCHL3* | 10.08 |
| 54165 | *RP42* | 56.64 | 10906 | *FLN29* | 7.30 |
| 54165 | *RP42* | 56.54 | 4287 | *ATXN3* | 7.12 |
| 27342 | *RABGEF1* | 44.54 | 51720 | *RAP80* | 6.25 |
| 8708 | *USP5* | 44.27 | 54472 | *TOLLIP* | 5.61 |
| 10422 | *UBADC1* | 22.83 | 54472 | *TOLLIP* | 5.35 |
| 10254 | *STAM2* | 22.29 | 2260 | *FGFR1* | 5.33 |
| 55040 | *EPN3* | 19.56 | 56852 | *RAD18* | 4.52 |
| 84959 | *STS-1* | 19.11 | 92815 | *HIST3H2A* | 4.16 |
| 78990 | *OTUB2* | 18.27 | 221302 | *C6orf113* | 4.14 |
| 8780 | *RIOK3* | 17.70 | 94107 | *MGC14327* | 4.08 |
| 51035 | *LOC51035* | 14.52 |  |  |  |
