# Supplementary material for: Diversified Application of Barcoded PLATO (PLATO-BC) Platform for Identification of Protein Interactions
Source: Genomics Proteomics Bioinformatics. 2019 Sep 5;17(3):319–31. doi: 10.1016/j.gpb.2018.12.010 (PMC6818353; doi:10.1016/j.gpb.2018.12.010)
Supplement: Supplementary Table S5 [file mmc6.docx]

**Table S5 Hit list of the PLATO-BC assays for ZIKV-NS5**

| **Gene ID** | **Gene name** | **Ratio (ZIKV-NS5/Pep)** |
| --- | --- | --- |
| 28974 | *HSPC023(C19orf53)* | 5.46 |
| 115098 | *LOC115098(CCDC124)* | 5.10 |
| 23429 | *RYBP* | 5.05 |
| 23429 | *RYBP* | 4.83 |
| 56288 | *PARD3* | 4.50 |
| 3622 | *ING2* | 4.37 |
| 55854 | *LEREPO4 (ZC3H15)* | 4.34 |
| 120534 | *FLJ38968 (ARL14EP, C11ORF46)* | 4.15 |
